# Liver fat and a perturbed metabolic milieu: a consilience of factors driving liver cancer development

**DOI:** 10.1007/s12072-022-10352-5

**Published:** 2022-06-13

**Authors:** Jacob George, Takumi Kawaguchi

**Affiliations:** 1 Storr Liver Centre, The Westmead Institute for Medical Research, Westmead Hospital and University of Sydney, Sydney, NSW 2145 Australia; 2 Division of Gastroenterology, Department of Medicine, Kurume University School of Medicine, Kurume, 830-0011 Japan

One of the prescient and incontrovertible outcomes of the global debate on MAFLD versus NAFLD has been to place the fatty liver disease we commonly see in clinical practice at the epicentre of systemic metabolic dysregulation. While the previous term non-alcoholic fatty liver disease (NAFLD) has been used for decades and the association with metabolic syndrome components well understood, the critical role of the dysregulated metabolic milieu was merely an association with an underappreciated pathogenic role. Within the conceptual framework provided by the metabolic (dysfunction) associated with fatty liver disease (MAFLD) name and definition both for adults and children [1–3], this pathogenic association has been recognised and has moved to the forefront. According to the current definition, the disease has to have the *sine qua non* of metabolic dysregulation, whether defined by obesity, type 2 diabetes (T2D) or at least 2 metabolic risk factors (elevated waist circumference, hypertension, dyslipidaemia, pre-diabetes, elevated homeostasis model assessment of insulin resistance score, or an elevated plasma high-sensitivity C-reactive protein level). Since then, a series of papers have indicated the utility of the definition for defining patients at high risk of hepatic (including liver cancer) and extrahepatic disease, the latter including cardiometabolic disease and cancer [4–6].

In this issue of the journal, Chen et al. [7] use a longitudinal nationwide cohort from Taiwan to assess the risk of incident liver cancer in patients with metabolic syndrome (MetS) alone, NAFLD alone, overlap NAFLD/MAFLD, and coexisting MetS and NAFLD. The primary cohort comprised individuals with both NAFLD and MetS based on ICD-CM-9 codes while the comparator groups (MetS alone, NAFLD alone, overlap NAFLD/MAFLD and individuals without MetS and NAFLD [control population]) were selected by propensity matching one to one by age, sex, index date and case number with no consideration to  hepatitis virology status or other risk factors. The mean follow-up period for the various groups ranged from 7.16–8.62 years with wide standard deviations. The salient findings were that patients with NAFLD alone, NAFLD/MAFLD, and coexisting NAFLD and MetS exhibited a 6.08-, 5.81-, and 15.33-fold higher risk of developing liver cancer respectively, after adjusting for age, sex, comorbidities and the Charlson Comorbidity Index. A similar risk between NAFLD alone and NAFLD/MAFLD is expected since they largely overlap, with NAFLD without MAFLD comprising between 5 and 10% of NAFLD cases. In contrast, those diagnosed with MetS alone did not have a significant excess risk of developing liver cancer compared to individuals who had neither NAFLD or MetS. Of relevance, the risk of liver cancer increased between 1.5 and twofold in patients with renal impairment, while that for cirrhosis increased three to sevenfold in the same groups after adjusting for age, sex and comorbidities. With regard to the age of liver cancer development, the proportion for groups aged 50–59, 60–69, and 70–79 years was 35.09%, 41.25% and 31.98%, respectively; ~ 80% of cancer cases were observed in individuals with both NAFLD and MetS who were 50 years of age or older. From this data, the authors conclude that metabolic dysregulation in the context of fatty liver disease has a significant impact on the risk of liver cancer development. Further, they suggest that liver fat and chronic hepatic inflammation (morphological or molecular) are a prerequisite in the MetS group for the development of liver cancer.

Because of the shear prevalence of fatty liver disease in both the developed and developing world approaching one in four adults [8], and with the worldwide burden of chronic viral hepatitis declining through vaccination or treatment, fatty liver disease is likely to become the commonest cause for primary liver cancer. In the Asian Pacific, NAFLD prevalence was estimated in a meta-analysis to be 27% [8], while the prevalence of MAFLD in those who are overweight or obese is about 50% [9]. As the paper by Chen et al. [7] highlights, for patients with NAFLD alone without concomitant MetS, the risk for liver cancer development increased ~ sixfold likely because they had one or more risk factors for metabolic dysregulation without meeting the MetS definition. Thus, the presence of metabolic dysfunction should be investigated at the time of diagnosis of liver cancer in any patient with fatty liver. While not examined in this study, the MAFLD definition also includes individuals with liver fat and metabolic dysregulation irrespective of the presence of other concomitant liver diseases including from the perspective of prevalence, chronic viral hepatitis B and C and alcohol-related liver disease. Since these other diseases, particularly (but not always) in the context of cirrhosis are associated with an elevated risk for the development of liver cancer, it behoves clinicians to be cognizant of the persistent risk of liver cancer development and liver disease progression in people with any fat accumulation in the liver and metabolic dysregulation, even if other liver diseases are cured or controlled. This is highlighted in a very recent study published on the ITA.LI.CA database [4]. In that study of 6882 cases of primary liver cancer followed at Italian liver centres, MAFLD was diagnosed in more than two-thirds of patients. Of most concern, single etiology MAFLD was expected to overcome hepatitis C-related liver cancer and mixed etiology MAFLD in about 4 and 6 years, respectively. According to their estimates, MAFLD HCC will become the only form of HCC in Italy in about 10–12 years. While in much of the Asian-Pacific chronic viral hepatitis and alcohol-related liver disease will dominate as a cause of liver cancer, the trend is unstoppable, with MAFLD in all its forms dominating the future landscape of primary liver cancer.

An interesting observation of the current study [7] was the further 2.5-fold increase in liver cancer development in fatty liver disease in the context of the metabolic syndrome defined by the ATPIII diagnostic criteria. This is to be expected given that those with the “full-house” are likely to have a worse metabolic milieu than those with only some of the diagnostic risk components. At a mechanistic level, the metabolic syndrome has pathologic correlates driven by excess adiposity resulting in hyperinsulinemia, insulin resistance, lipotoxicity, inflammatory cytokines release and innate and adaptive immune effects among others. These factors not only converge on the liver to increase the risk of cancer development and liver disease progression but equally have extra-hepatic effects driving cardiometabolic disease and extra-hepatic cancers in a deleterious interactome. In this regard, a very recent report of hepatic, adipose and blood lipidomics suggests that fat accumulation in the liver is associated with the generation of lipotoxic lipid species including diacylglycerols, and shingolipids, including ceramide that are reflected in plasma [10]. It indicates that liver lipid is toxic not only to the liver but importantly, also in a systemic context. In contrast, the adipose lipidome did not correlate with the liver lipidome. Another interesting observation from the above study was that there were no qualitative or quantitative changes in liver lipids with the development of steatohepatitis in comparison to livers with steatosis highlighting that the mere presence of liver lipid in the context of metabolic dysregulation is toxic to the liver and to the systemic compartment [10].

An intriguing observation is that metabolic syndrome per se did not increase the risk for liver cancer development in the absence of liver fat. This finding is against the overwhelming weight of data including meta-analyses [11] suggesting that metabolic dysregulation as encapsulated in the various operational definitions of MetS not only increases the risk of primary liver cancer but also a variety of other extra-hepatic cancers including those of the gastrointestinal tract, lung, breast, gynecological and urinary system. The authors postulate that it could be that previous studies might not have excluded the presence of liver fat or hepatitis in their cohorts. However, decades-old data, replicated in many studies suggest that up to 90% of people with fatty liver disease meet the definition of MetS [12] and it is accepted that liver fat is the hepatic manifestation of this syndrome. For individuals to have MetS and insulin resistance, without the presence of liver fat—a cardinal and early manifestation—would be very unusual. Further, in their own analysis [7], the adjusted hazards ratio for incident liver cancer was increased for individual MetS components including obesity (3.4-fold), hypertension (7.26-fold), dyslipidemia (2.64-fold), and T2D (11.82-fold), consistent with previous literature. Their findings, therefore, are intriguing and not immediately reconcilable given the known pathophysiology of MetS. Uncoded fatty liver disease in their cohort with MetS only would in fact if anything, increase the risk of liver cancer development. Perhaps their observations (based on diagnostic health records coding, with its inherent limitations) selected for the small minority of individuals in whom MetS was present without its usual hepatic accompaniments, in which case the substrate for cancer development would not be present. In this regard, it would have been interesting to analyse if MetS was associated with extra-hepatic cancers in their cohort.

Finally, it should be borne in mind that like all meta-data studies derived from electronic health records, the quality of the outcomes is based on the quality of the coding which reflects the disease that is the subject, in this case fatty liver disease. In this regard, only 6724 individuals with incident fatty liver and MetS comprised the study group after a mean follow-up in excess of 7 years from an initial population of 1 million adults [7]. While the data is the data, to this reader, the incidence appears lower than expected given that a recent meta-analysis from Asia suggests a pooled annual NAFLD incidence rate of 50·9 cases per 1000 person-years [13]. It should also be noted that coding has its inherent limitations. For example incident fatty liver disease was diagnosed using ICD-9-CM codes 571.40, 573.3, and 571.8. Code 571.40 codes for chronic hepatitis unspecified, while 571.8 codes for other chronic nonalcoholic liver disease and 573.3 for hepatitis unspecified.

**Fig. 1:**
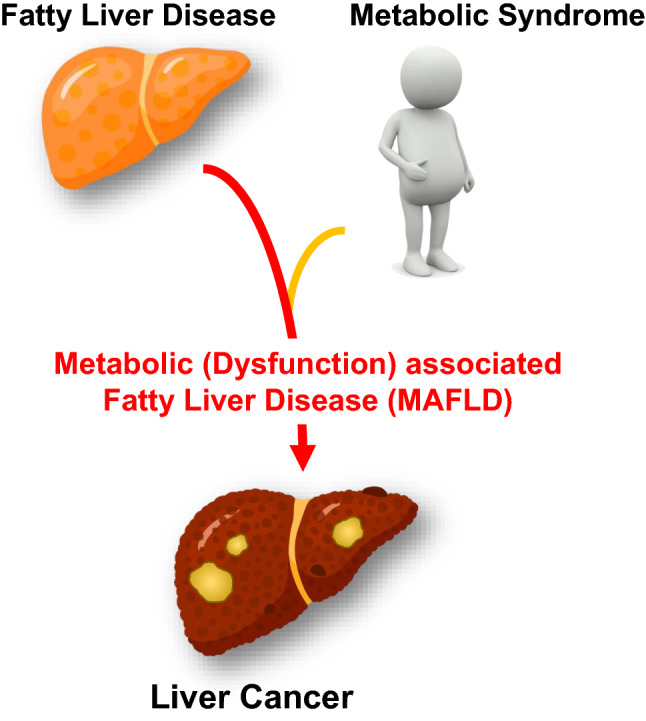
The presence of systemic metabolic dysregulation typified by metabolic syndrome in patients with fatty liver disease results in metabolic (dysfunction) associated with fatty liver disease (MAFLD) and increases the risk of liver cancer

Despite these caveats, Chen et al. [7] have provided robust data from Asia on the central role of systemic metabolic dysregulation and its impact on incident liver cancer. Part of this milieu is the presence of liver fat which in the context of the company it keeps (systemic dysregulation in homeostatic mechanisms) aggravates liver cancer risk. Given the unequivocal and rising body of worldwide data supporting the association of liver fat in conjunction with MetS components such as obesity and diabetes in increasing the risk of liver cancer, at the individual patient level hepatologists need to move towards treating both the liver fat and the components of the MetS for a more holistic approach to patient care rather than just focusing on the liver. Better still, disease prevention or mitigation through lifestyle intervention should be the core of our approach to these individuals (Fig. 1).
